# Multiple cranial nerve deficits as preceding symptoms of systemic non‐Hodgkin’s lymphoma

**DOI:** 10.1111/cns.13097

**Published:** 2019-01-01

**Authors:** Jing‐Jing Li, Bao‐Shan Qiu, Jia‐Xin Chen, Da‐Wei Liu, Shi‐Hui Xing, Hong‐Bing Chen, Jin‐Sheng Zeng, Hui‐Yu Feng, Yu‐Hua Fan

**Affiliations:** ^1^ Department of Neurology and Stroke Center National Key Clinical Department and Key Discipline of Neurology The First Affiliated Hospital Sun Yat‐Sen University Guangzhou China; ^2^ Department of Pathology The First Affiliated Hospital Sun Yat‐Sen University Guangzhou China

Multiple cranial nerve deficit is a common neurological manifestation. It is usually presented in schwannoma and meningioma patients but rarely in lymphoma.^1^Non‐Hodgkin’s lymphoma accounts for 90% of lymphomas, and its clinical manifestations differ with diverse primary sites.^2^ Approximately 5%‐10% of systemic non‐Hodgkin’s lymphoma is characteristic of central nervous system invasion, such as secondary central nervous system lymphoma.^3^ Multiple cranial nerve deficits are rarely the primary manifestation of non‐Hodgkin’s lymphoma. In this study, we aimed to prove that multiple cranial nerve deficit can be onset symptom of non‐Hodgkin’s lymphoma. In addition, neurologists should pay more attention to the diagnosis of lymphoma in the patients with cranial nerve deficit.

We retrospectively analyzed the clinical, radiological, pathological, and cerebrospinal fluid features of five cases with primary manifestation of multiple cranial nerve deficits who were admitted to the neurology department, the first affiliated hospital of Sun Yat‐sen University. The clinical features included symptoms, diagnosis and treatment history, and prognosis. Radiological results included cranial magnetic resonance imaging (MRI) and positron emission tomography‐computed tomography (PET‐CT). Cerebrospinal fluid (CSF) examination included pressure, white blood cell count, protein content, cytology, and flow cytometry; and pathological classification was conducted according to the 2016 revision of the World Health Organization (WHO) classification of lymphoid neoplasms.^4^ The study protocol has been approved by local committee on human research, and we got informed consent of the patients.

The average age of the patients was 46 years old (range from 24 to 67),with 2 males and three females. The patients’ clinical, radiological, and cerebrospinal fluid features are shown in Table 1. Symptoms of multiple cranial nerve deficits of the patients involved blurred vision, diplopia, ptosis, facial palsy. The 3rd and 6th cranial nerves were the most commonly impaired. Cranial MRI showed cranial nerve and leptomeningeal involvement in five patients without parenchymal lesions. The cerebrospinal fluid (CSF) white blood cell and protein levels of three patients were significantly increased. Cytological examination of the CSF revealed the presence of tumor cells. All of the patients were ultimately diagnosed as non‐Hodgkin’s lymphoma by pathological biopsy (Figure 1), of which four were B‐cell lymphoma (three cases of diffuse large B‐cell lymphoma (DLBCL) and one case of Burkitt’s lymphoma) and one was T‐cell lymphoma. All patients exhibited secondary involvement of the CNS. While in hospital, all patients worsened progressively. Cases 2, 4, 5 refused aggressive treatments and then died for progressive whole‐body deterioration within a month of discharge, Case 1 died 6 months after chemotherapy, and Case 3 lacked follow‐up.

**Table 1:** The patients’ clinical, radiological, and cerebrospinal fluid features

Case	Multiple cranial nerve deficit range	MRI	PET‐CT	Cerebrospinal fluid				
				pressure	Leukocyte	Protein	Presence of tumor cells	
1	IIIIIVII	Cranial nerve thickening and enhancing, tentorial meningeal thickening and enhancing	Active metabolism in multiple parts of the body, for example, neck lymph nodes, meninges, breasts, gastrointestinal system, and other locations	&gt;300	2120	2313	Yes	
2	IIIVVIIXX	Frontal temporal meningeal thickening involving the left cavernous sinus	Active metabolism in multiple parts of the body, for example, breasts, peritoneum, iliopsoas, bone, and other locations	265	1100	699	No	
3	IIIIIV	Maxillary sinusitis, sphenoid sinusitis	‐	‐	‐	‐	No	
4	IIIVII	No obvious abnormalities	‐	125	60	1407	No	
5	III VI	Cranial base nasopharyngeal carcinoma, invasions on the vessels and nerves of the left cavernous sinus area	‐	‐	‐	‐	No	

**Figure 1:**
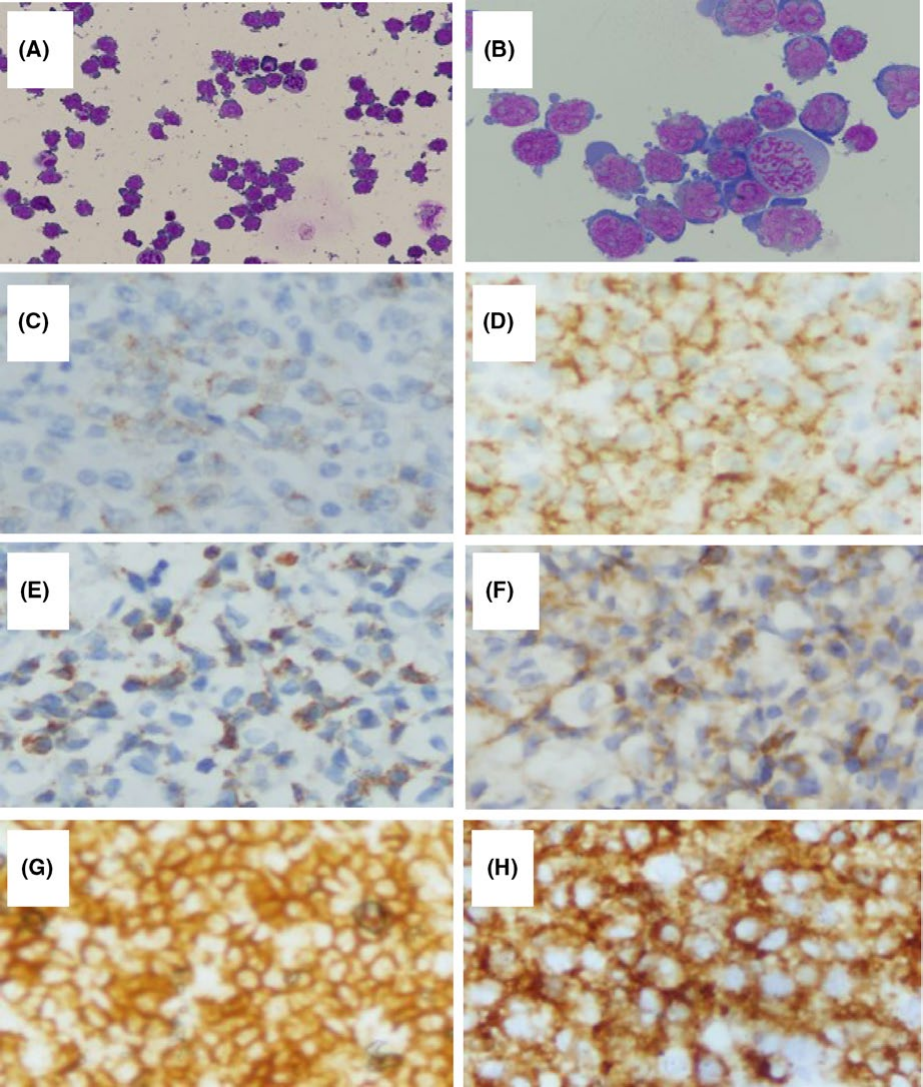
Immunohistochemical staining (IHC) of biopsy results and cerebrospinal fluid cytology results. **(**A, B) cerebrospinal fluid cytology May‐Grunwald‐Giemsa(MGG) staining of case 1, showing abnormal lymphocytes(A,MGG, ×400;B,MGG, ×1000). (C, D) case 3, Tumor cells show strong expression of CD20and CD79a (IHC, ×400). (E, F) case 4, Tumor cells show strong expression of CD3 and CD43(IHC, ×400). (G, H) case 5, Tumor cells show strong expression of CD20and CD79a (IHC, ×400)

Our study showed the multiple cranial nerve deficits can be the mainly or primarily manifestations of non‐Hodgkin's lymphoma patients. We clarified the clinical and pathological characteristics of these patients and suggest that neurologists should pay more attention to the diagnosis of lymphoma in those patients even without parenchymal invasion. Tumor is a common cause of multiple cranial nerve deficits, frequently involving schwannoma and meningioma but rarely lymphoma.^1^ Systemic non‐Hodgkin’s lymphoma patients with systemic neurological symptoms point to the diagnosis of secondary central nervous system lymphoma (SCNSL).^5^ Hyperintensities in parenchyma, cranial nerves, and pia matter are common signs in the contrasted MRI of patient with SCNSL. It can be easily misdiagnosed as inflammatory diseases. Pathological biopsy should be conducted on highly metabolic areas in these patients to make accurate diagnosis. The timing of the biopsy and the detection efficiency also affect the accuracy of diagnosis. The cytology of CSF is very important for the diagnosis of SCNSL.^6^ The morphology of lymphoma cells is an important part of CSF cytology and the primary basis for the diagnosis. Furthermore, it is necessary to integrate the findings of cytochemical staining and flow cytometry to provide more objective evidence.

Cranial nerves involvement could be related to many causes such as CNS inflammation, brain tumor, cerebrovascular disease, craniocerebral trauma, infection, and diabetic multiple cranial nerve deficits. And it is a rare onset symptom in lymphoma patients. In these patients, cranial nerves involvement can be easily misdiagnosed as diseases as mentioned above. CNS infiltration is a rare but highly lethal complication of malignant lymphoma, with a median survival of only 4‐5 months.^7^ The occurrence of CNS invasion in DLBCL is rather rare. Ferreri et al found that CNS invasion was present in 2‐5% of DLBCL patients after diagnosis or during progression and relapse.^8^ The patients with CNS invasion prior to lymphoma diagnosis had significantly longer survival time.^9^ Proved standard therapies on SCNSL are limited, in patients with SCNSL, longer survival has been observed, particularly when high‐dose methotrexate (HDMTX)‐based chemotherapy was administered.^10^ Only Case 1 who had a longer survival time underwent chemotherapy and HDMTX after diagnosis.

In conclusion, it is important for a neurologist to pay attention to the diagnosis of lymphoma when patients manifest with multiple cranial nerve deficits as primary or main clinical symptoms. It requires the integration of knowledge from neurology and hematology. In general, cytology of the CSF is important for diagnosis, and PET‐CT examination can be used to clarify the degree of involvement of the entire body, while the biopsy of pathological tissues is the gold standard for diagnosis.

## CONFLICT OF INTEREST

The authors declare no conflict of interest.
